# Transnational Migration and Dual Career of Slovenian and Swiss Elite Female Handball Players—A Longitudinal Analysis

**DOI:** 10.3390/sports10090137

**Published:** 2022-09-16

**Authors:** Marta Bon, Mojca Doupona, Susan Wilson-Gahan, Laura Capranica, Flavia Guidotti

**Affiliations:** 1 Faculty of Sport, Department of Sport Sociology, University of Ljubljana, 1000 Ljubljana, Slovenia; 2 European Athlete as Student (EAS) Network, GXQ 1025 Ghaxaq, Malta; 3 Faculty of Business, Education, Law and Arts, University of Southern Queensland, Toowoomba, QLD 4350, Australia; 4 Department of Movement, Human and Health Sciences, University of Rome “Foro Italico”, 00135 Rome, Italy

**Keywords:** sport migration, dual career paths, women handball, qualitative analysis, relocation challenges

## Abstract

The purpose of this study was to identify the career paths of transnational migrating female elite handball players. Fourteen Slovenian and Suisse national team players were monitored over a 7-year period by means of semi-structured interviews and official handball records. At the end of the examination period, six still-active players were interviewed again. Qualitative thematic analysis was employed to develop a contextualized understanding of participants’ careers paths and life trajectories in relation to their athletic migration and dual career. In relation to the limited opportunities offered by small countries with middle-ranking national handball teams, participants highlighted that sport migration coupled with dual career opportunities represented a strategic decision for a successful career development through several key factors: (1) a clear intention towards a professional handball career; (2) the actual fulfilment of professional handball career aspirations; (3) dual career goals as part of the migration process; (4) high personal ambition and emotional connection to handball; (5) the implementation of a successful dual career path; (6) a positive migration experience; and (7) feeling supported and valued during relocation. Sport federations and elite clubs should consider the implementation of a multidimensional approach encompassing dual career paths to facilitate athletes’ transnational relocation and career transitions.

## 1. Introduction

Globalization is a process encompassing the causes, the course, and the consequences of transnational and transcultural integration, consolidating the world into a whole space for individual progress in many sectors of the society at large, including sport [1,2,3]. In fact, extended cross-national networks may represent a springboard for contemporary athletic career development, with transnational mobility of sportspersons (i.e., youth talented athletes, senior top-level athletes, and elite coaches) presenting a relevant opportunity for the individual’s personal and professional growth [2,3].

In recent decades, a growing number of athletes and sports professionals engaged in transnational sport migration and/or relocation within their native country to foster their athletic development, to expand their professional networks, and/or to strengthen their dual career paths (i.e., combining elite sport with academic and/or vocational education) [2,3,4,5,6,7]. Furthermore, athletes’ life and career decisions might be also impacted by their identity orientation (e.g., National; Transnational; Multinational; Post-national; Intra-national; Non-national) and willingness to engage in athletic mobility, characterized as a dynamic, complex, and fluid personal process [8]. In particular, transnational migrant athletes experience a multiplicity of interactions and relations (e.g., social, cultural, political, sport-specific) in the relocating countries [9]. In this framework, both short-term mobility (i.e., typically involving an acute adjustment to a new cultural context) and long-term migration (i.e., encompassing a longer and more structured adaptation and integration processes), have been both found as strategic career advancement factors [7,9].

A sports career is a dynamic, multidimensional, multilevel, and multifactorial process, in which the athletes’ efforts, dual career obligations, and socio-cultural and life aspects affect the transition from youth talent to the mastery elite level, and post-athletic career retirement [10,11,12,13,14,15,16]. In progressing along their developmental continuum, athletes experience increases in training/competition schedules and time-consuming journeys, possible injuries, and long-lasting recovery [17]. To overcome the demands of an elite sport career, athletes need to develop strong competencies, skills, attitudes, knowledge, and specific experience [5,18,19,20,21]. Furthermore, the external socio-cultural environment, resources, and support structures play a crucial role in facilitating or constraining athletes’ career paths [18,22], also in relation to their gender [2,21,23,24,25]. In this framework, athletes could consider sports migration across European countries or wider geographical areas as a potential opportunity of personal and professional growth, mirroring the individual’s desire to ameliorate his/her own status [2,3,5,26]. Border crossings can be understood as critical and cultural turning points, or transitions, in the athletes’ careers [27]. In particular, cross-national mobility underpins communication in a new language and the individual integration into a new culture, as well as different sport-specific and/or national patterns/models of career progression, which are substantial determinants of athletes’ decisions and career satisfaction in relation to their gender, nationality, race, values, and religion [2,5,9,28].

According to the literature [2,3,29,30], specific stressors and challenges for migrant athletes have been identified. Cultural integration and linguistic adaptation represent additional demands to other well documented structural barriers to sport and/or academic adaptation and progress [2]. Sport-related challenges of fitting into the training schedule/routines and the playing style of a new team also proved to be central in players’ experience, feelings, and perceptions about athletic migration [30,31]. Moreover, these aspects influence the development of a sense of belonging in relation to the sport environment [5,32,33,34,35]. The literature also pointed out the essential role of the family, friends, peers, and supporters in facilitating and/or constraining the athlete’s migration process [2,11,34,36,37].

Research on athletic labor migration has investigated athletes’ motivations and reasons for sport migration [2,3,4,38,39,40,41,42]. Some studies have highlighted that the motivating and demotivating experiences of migrant athletes are intertwined with their adaptation processes, governmental and/or sport federation’s interventions, financial resources and support, and integration strategies within the receiving sports context [28,43]. According to Ryba and colleagues [33], the athletes’ migration and/or relocation are characterized by three main phases: (i) a pre-transition stage, which refers to psychological aspects related to job mobility involving various ways of planning for future relocation and for psychological disengagement from the athlete’s native country; (ii) an acute cultural adaptation stage, during which athletes attempt to navigate, negotiate, and evolve their own understandings within the cultural patterns of the receiving environment. Thus, migrating athletes might experience feelings of loss and loneliness, or excitement and hope for new opportunities; and (iii) a socio-cultural adaptation stage, characterized by long-term stay or permanent settlement in the receiving country.

In general, a sport career might span over 15 years from the talent development to the elite senior levels; sport migration might occur at a different career stage and/or athlete’s age, with specific national and sport-specific aspects playing a modulating role [3]. Furthermore, sports careers often occur parallel to academic careers, and their combination (i.e., dual career) is considered a fundamental aspect of the holistic development of the athletes, which prepares them for a professional life after their athletic retirement [44,45,46]. In considering that transnational migration could occur when the athletes are enrolled in their academic paths, opportunities for dual career adjustments could be considered crucial [2,5,21,47,48,49]. In the United States where dual careers are well structured, six typologies of migrating student athletes have been identified [50]: (1) Mercenary, an athlete motivated by the economic support (i.e., scholarships); (2) Settler, an athlete settling in another country for a prolonged period, also beyond the competitive career; (3) Returnee, an athlete moving back to their native country at the end of their dual career; (4) Nomadic Cosmopolitan, an athlete desiring to experience different countries and cultures; (5) Ambitious, an athletes wishing to pursue a professional sport career through a good quality dual-career environment; and (6) Exile, an athlete migrating for personal or political reasons. In Europe, where a dual career is characterized by country-specific and cultural–organizational regulations in the field of sport and education [51], a systematic literature review on European dual career migrating athletes identified three categories [3]: (1) Mercenary, athletes valuing the possibility to combine high-quality academic and education paths; (2) Ambitious, athletes wishing to pursue a sport career in a high-profile country in relation to their sports discipline; and (3) Exile, an athlete wishing to live abroad.

In athletic migration, one important modulating factor concerns the gender of the athletes [25,52]. In recent decades, gender mainstreaming and the progressive growth of women’s sports have been observed [53]. However, many sports disciplines still present unequal gender-related career trajectories, outcomes, and professional recognition (i.e., professionalism vs. amateur level; unequal payment; unequal portrayal on the media), with female athletes receiving less opportunities with respect to their male counterparts [53,54]. Furthermore, few studies investigated European migrating dual career female athletes [3]. Finally, gender differences in athletic career paths might be also affected by the relevance of the sport disciplines within national contexts, which might determine higher or lower motivations of athletes towards relocation and/or transnational migration. It has been also reported that female athletes need to financially support themselves more often than their male counterparts, being obliged to combine their sport career with additional working duties [2,49].

In handball, differences in cross-national relevance of this team sport may influence the career trajectories of sportspersons [7]. Players and coaches from countries holding a low position in the international handball ranking might consider migrating to pursue their professional careers in top-level ranked countries offering high-level training conditions, competition levels, economic rewards, sponsorships, and media coverage [7,55]. In this framework, the aim of this study was to develop a contextualized understanding of the sport migration of elite female handball players from geographically small countries presenting low professional prospects in this specific sport, also in relation to their personal motivations, dual career, and post-athletic career perspectives.

## 2. Materials and Methods

### 2.1. Qualitative Approach to the Problem

For the present study, a 7-year (from 2012 to 2019) longitudinal qualitative approach was applied to investigate migration experiences of elite female handball players members of the Slovenian (SLO) and Switzerland (SUI) National teams, which held a middle-rank European position during the examination period (SLO: range 10th–12th; SUI: 21st–24th). In particular, the following inclusion criteria have been adopted for both the cross-sectional analysis at the beginning of the study and the longitudinal analysis: (i) being an elite athlete and active member of the handball national team in the native country; (ii) being a sport migrant; and (iii) being enrolled in a dual career path at the beginning of the experimental period. Thus, quitting the sport career, and/or lowering the competition level, and/or losing the “sport migrant” status during the experimental period were considered as exclusion criteria for the longitudinal analysis.

Qualitative thematic analysis has been applied to develop a contextualized understanding of the career and life trajectories of female handball players during their migration experience, including the management of their dual career. In this framework, the present study intended to integrate and expand previous research findings regarding cross-national mobility in elite athletes [2,3,5,9] with qualitative knowledge regarding major migration characteristics of women elite handball athletes migrating to pursue better career opportunities with respect to those they might have experienced in their native country. In particular, the following research themes were considered crucial: (i) the effect of elite women handball athletes status in the athlete’s native country on the personal decision to migrate; (ii) the organization of the migration; (iii) the role of the family in the migration process; (iv) the challenges faced in the relocation process, including the adaptation experiences in different socio-cultural settings; (v) the sport and academic perceived success during migration; and (vi) the post-athletic career opportunities after sport migration.

According to the literature [56,57], semi-structured interviews have been considered appropriate to elicit participants’ perceptions and opinions in relation to their sport migration experiences. To validate the semi-structured interview’s content, it is relevant to consider the assessment made by various experts on different aspects of the interview. Profiting from an extensive knowledge and proven experience in the area of the dual career of the sportspersons and the international handball community, the research team engaged in a focus group to develop the semi-structured interviews and reached a consensus on the clarity of the content, clarity of the wording, and the grouping of questions deemed relevant to provide the necessary data to obtain the information relevant to the objectives of the study. Thus, guidelines for the conduction of the semi-structured interview, and the analysis and the interpretation of results, were developed.

### 2.2. Participants

Fourteen female handball players from SLO (*n* = 8, aged 23.2 ± 6.5 years) and SUI (*n* = 6, aged 24.9 ± 4.2 years) national teams presenting the “elite migrating dual career athlete” status volunteered for the present study. The sample represented the most successful female handball national team players in both countries, having played more than 50 national team matches.

### 2.3. Interviews and Procedures

The semi-structured interview framework encompassed questions covering the major research themes and follow-up questions based on participant’s responses, allowing space for individual verbal expressions [57]. In particular, athletes were asked to recall their dual career development (i.e., including sport results, educational achievements, and challenging career transitions), reasons to engage in a sport migration process (i.e., including migration pattern, perceived challenges, adaptation experiences in different socio-cultural settings, and career outcomes after relocation), the impact of their career trajectory on other personal/life domains, (i.e., including their relationships, the role of the family in their migration experience and post-athletic career prospects), and post-athletic career opportunities derived from cross-national mobility.

To confirm the coverage and relevance of the interview content and structure, and/or to identify the possible need to reformulate and/or implement questions, a preliminary version of the instrument was piloted through internal testing within the research team [58], which reached a consensus agreement during a focus group. After the preliminary testing phase, the tool was considered suitable to be applied in the research setting.

The longitudinal research design of the present study was characterized by a four-phase data collection:

1. The first phase (in 2012) was for the recruitment of the SLO and SUI women handball athletes during a national team preparation camp, based on inclusion criteria. Athletes were informed about the aim and procedures of the study, the confidential nature of the data collection, and the possibility to drop the research at any time with no explanation. To voluntarily enroll in the study, all the players provided their written informed consent and general demographic information. Thus, data were coded to guarantee the anonymity of the participants.
2. The second phase (in 2012) was for the administration of the first semi-structured interviews (“Interview 1”) to the research sample. At this research stage, an emphasis was placed on the athletes’ perceptions regarding their current sport and academic status, and the reasons for sport migration. Interviews were arranged at a time and in a place convenient to the athletes and conducted in-person by a research team member.
3. The third phase (from 2012 to 2019) consisted of a 7-year monitoring period of the athletes’ career development, characteristics of their migration process (i.e., permanency in the destination country and/or further relocations), sport-specific information (i.e., national team matches, changes in the national team squads, professional activity and achievements with their destination club as migrating athletes), and dual career paths. Contextual data were collected both through individual contacts with the involved players, and from the SLO and SUI National and European Handball Federation on-line platforms.
4. The fourth phase (in 2019) consisted of a second semi-structured interview (“Interview 2”) administered only to the participants still presenting the “elite handball migrating athlete” status. Conversely, athletes not meeting the inclusion criteria have been excluded from this research stage. To check the quality of the collected responses at the Interview 1, to verify the accuracy of the information retrieved in the monitoring phase (i.e., migration in new clubs, playing performance, scoring results, national team performance, and dual career activities), and to stimulate further reflection on major career events occurred during their migration experience, “Interview 2” started with athletes cross checking the previous research material. In this phase, an emphasis was given to the shifting discourses regarding athletes’ insights and personal evaluation regarding their career and life journeys, as a result of their migration choices. Interviews were conducted both face-to-face and online during the season 2019/2020.

The interview duration ranged from 50 to 105 min for each athlete. By considering the different nationalities of the participants in the present study, in order to allow athletes to freely express themselves and to encourage them to provide comprehensive perceptions regarding their career development and migration features, the semi-structured interviews were conducted in their native language (i.e., Slovenian and German languages for SLO and SUI athletes, respectively). To refine the accuracy and quality of the collected athletes’ responses, interviews have been recorded and transcribed. Subsequently, the final text of each interview was translated into English and reviewed for language correctness by a native speaker (i.e., a member of the research group) before data processing and analysis.

### 2.4. Data Analysis

To identify major participants’ career development patterns, Table 1 reports the thematic analysis in relation to the career trajectories of migrating elite female handball athletes. According to the literature, the analysis encompassed 7 stages [59,60,61]. In the first stage, the final English version of semi-structured interviews transcripts and collected longitudinal information was aggregated for each individual athlete. In the second stage, a familiarization with collected data was performed, proceeding through initial readings of the transcripts to find the most salient aspects in the participants’ testimonies. During the third stage, a preliminary coding system was generated, grouping elements of data according to similarities or perceived patterns. During the fourth stage, the search for major thematic domains was realized, grouping coded data into major clusters according to similarities and/or patterns. In particular, three main thematic areas have been identified: (i) “Dual career”, which pertained major features in relation to athletes’ sport and educational career development, including the athletic and academic organization, challenges, and performance in relation to their migration; (ii) “Personal/life sphere”, concerning the individual motivations and expectations in relation to athletic migration, the impact of the mobility on the athletes’ personal growth, and the role of the family/peers/agents in supporting their relocation; (iii) “Migration”, including the identification of specific features regarding the organization, barriers, challenges, and socio-cultural adaptation strategies of athletes in relation to their cross-national mobility. For the fifth stage, migration phases (i.e., before, during and after migration) were selected as subthemes to organize coded data and to provide a chronological comprehensive overview of the athletes’ migration process. In the sixth stage, the accuracy of the entire material was assessed to ensure that themes, subthemes, and codes were clearly delineated and appropriately positioned. Finally, the seventh stage encompassed the final review and analysis of the whole material.

## 3. Results

### 3.1. Before Migration

Table 2 presents the athletes’ information prior to migration. All the players reported to have started playing handball during childhood, with parents and teachers playing a crucial role in initiating their sport career. With respect to their SUI counterparts, SLO players also highlighted the role of physical education teachers, who facilitated their early approach to handball at the elementary school level. Then, all the players experienced similar sport career transitions and development, from youth to top international levels as national team members.

Regarding the domestic relocation prior to transnational migration, with respect to SLO athletes SUI players tended to have a more static national sport career (i.e., long-term enrolment in the same sport club) due to the lack of perceived opportunities and prospective athletic growth resulting from relocation within the national handball context. In fact, domestic mobility was limited by the general amateur organization of SUI sport clubs, which was perceived as lacking potential opportunities for handball career progression. Conversely, national athletic relocation emerged as a common practice in Slovenia, where athletes tend to play for different clubs to ameliorate their athletic status. Finally, both SUI and SLO players reported 2–8 years of national senior elite experience before engaging in transnational athletic migration, which was deemed a necessary step for further career development. In fact, amateur and semi-professional athletes decided to migrate to pursue a professional sport career, with SLO athletes presenting a longer international experience at the beginning of this study with respect to their SUI counterparts.

The first interview in 2012 highlighted a lack of opportunities for a handball career development in the native country as the major reason for sports migration, which was perceived as a strategic choice to attain better sport conditions (i.e., competition level, internal sport club organization, quality of the coaches, training facilities, salary, visibility, etc.), dual career opportunities, and to increase competencies and life skills to prepare for post-athletic career retirement (i.e., professional growth, learn a foreign language). Family and peers were perceived as influencing both the athletes’ sport and personal development in their home country and their decision to migrate. In particular, transnational mobility was perceived as one of the most important stages in the athletes’ sport career development. Furthermore, relocation was associated with prospective athletic success, and improved quality of life and financial condition. Whilst sport migration represented the first opportunity for SUI athletes to be paid for playing handball, increased salary opportunities became a contributing and relevant factor for the migration decision for SLO players. In fact, SUI athletes valued the potential improvement of their handball skills during migration, which could contribute to ameliorate their engagement with the national team. Conversely, a perceived prospective enhancement of the social status emerged as a crucial factor for the transnational migration of SLO athletes. Additionally, access to dual career opportunities during relocation was reported a migration pre-requisite for the majority of the athletes.

In particular, the players highlighted:

*“My parents recognized very soon that I was more motivated for school when I was also doing well at handball. Although they had different plans for my future (a career in business), they supported me during my handball career”.*

(SUI player)

*“My mother was repeating to me, ‘You should go abroad. There everything is better than here: clubs are more structured and better organized, and salaries are better. If you invest so much time in all the stages of your sport career, why not getting economic benefits from handball? In addition, you will have better opportunities abroad. I would like to have you at home, but I wish you a better life; better than I have’”.*

(SLO player)

*“What really mattered for me and my parents was to be recognized in prominent foreign handball clubs; and, even more, to find a job abroad. The career goals were included in my sport and life plans from the beginning, being clear that I would migrate one day to change my life prospects”.*

(SLO player)

*“I was thinking about leaving the previous club in my home country for a while. I felt that my home club was not the right environment, at the same time I was not happy at home. My father was putting additional pressure on me with his permanent presence in my handball and in my life in general. I simply wanted to move”.*

(SLO player)

*“For me a key point was to migrate. My position in the family and my social status had turned drastically after migration. When I was playing at home, for my family I was “just another female handball player”; after I migrated, my social status was much higher—I was “someone”, the successful person, the family member to be proud of”.*

(SLO player)

Regarding the organization of the transnational migration, all the players reported that they prepared physically and mentally to cope with relocation challenges. Only three elite athletes with outstanding handball skills were directly contacted by foreign clubs, whereas difficulties in finding a good foreign club emerged for the rest of the players. However, the majority of the athletes stressed that being a national team member played a crucial role in their opportunities to migrate, since they “became visible” at the national team tournaments.

### 3.2. During Migration

Table 3 reports the athletes’ sport status and migration characteristics from 2012 until 2019. In general, 71% of the sample (*n* = 10; SUI: *n* = 3; SLO: *n* = 7) competed at an elite level throughout the examination period, whereas the remaining 29% athletes (*n*= 4; SUI: *n* = 3; SLO: *n* = 1) ended their sport career before 2019. Regarding migration, only 43% athletes (*n*= 6; SUI: *n* = 1; SLO: *n* = 5) showed an “elite sport migrant” status in 2019, which was retained till Interview 2. Conversely, in 2019, five athletes were still active but relocated in their native country. Only 36% of the sample (*n* = 5; SUI: *n* = 1; SLO: *n* = 4) maintained transnational migration during the 7-year study, whereas intermittent mobility was observed in five players, who experienced a period of relocation to their native country. At the end of the study, only one SUI athlete was still a top-level migrant player, whereas two athletes relocated to Switzerland and three retired at around 30 years of age, transitioning into the labor market and starting their family. Regarding SLO players, in 2019, only two athletes were top professional players participating in the Handball Champions League, and scored among the top players in Europe. Conversely, the other active SLO players moved in national and international semi-professional leagues and only one athlete retired before the end of the study.

Regarding the countries of migration, Germany was the most frequent destination for athletes (*n* = 8; SUI: *n* = 5; SLO: *n* = 3), followed by Spain (*n* = 4; SLO: *n* = 4) and France (*n* = 3; SUI: *n* = 1; SLO: *n* = 2). In particular, migration dynamics was more stable in the SUI sample with respect to the SLO one. In fact, the majority of the SUI athletes (*n* = 7) mostly played in the German league, probably due to the same native language. Conversely, SLO players presented a higher variety of destinations and reported a total of 20 between-country and 28 between-club sporting transfers.

As migrating athletes, the players tended to perceive a higher fear of injury with respect to their previous national experience, especially when a previous injury was experienced during their career. In particular, some athletes highlighted that the intensity and volume of a sport season were extremely challenging, due to the limited resting time between the regular season with their handball club and their commitment to the national team. By considering health determinants to maintain a professional athletic profile and for further sport career development, injury prevention and treatment represented an additional demand in the athlete’s daily schedule.

*“I can only remember that I was so scared after the injury. I was afraid of a new injury and of performing badly [again]. I was doing special mental training to relieve the fear of another injury, but I nevertheless felt under pressure all the time”.*

(SLO player)

Individual coping strategies with multiple challenges derived by an international sporting career have been emphasized as a key factor in determining career transitions outcomes. Having moved abroad, some athletes reported a variety of feelings, ranging from big expectations to substantial doubts regarding their life choices. Some experienced homesickness and wish to return home, especially when the sport and living conditions in the foreign country did not meet their pre-migration expectations. In this framework, perceived social support from parents, peers, teammates, and coaches played a crucial role in athletes’ adaptation to the new socio-cultural environment. Whilst many athletes reported a strong social support from family members during the whole migration period, other declared that their partners were mostly unwilling to adjust their lives to the athletes’ careers. Finally, language barriers affected the life and career management of migrating players who needed to learn a foreign language.

*“When I decided to move, I was really excited… but being abroad alone wasn’t so much fun in the beginning. I felt loneliness”.*

(SUI player)

*“In the beginning, it was very difficult to communicate with the coach or teammates, who were speaking French. Some of them helped me with translation in English, during practicing and playing matches; however, it was hectic with this communication. When I mastered the French language, I felt very happy and this had led to a better communication in handball”.*

(SLO player)

Regarding dual careers, the majority of the athletes enrolled in distance academic courses and graduated during their migration period. In particular, six athletes graduated in sport-related studies: one in social sciences, two in economics, and three became teachers. While abroad, after graduation six SLO athletes reported that they were combining their handball and vocational commitments.

Although financial aspects were not considered a key priority for migrating, especially SUI players appreciated the economic benefits deriving from their international athletic mobility.

*“It is not a lot of money, but I’m happy with that. I’m realistic. I know that I am not a world—class player or a handball star, so it is about what I can get. The experience counts more than [the] money I earn”.*

(SUI player)

### 3.3. After Migration

As displayed in Table 3, only six athletes (SUI: *n* = 1; SLO: *n* = 5) maintained the eligibility criteria for the longitudinal analysis and were interviewed in 2019. In fact, in 2019 they reported an overall average international experience of around 9 years, having started their international mobility before the beginning of this study. After such a long migration period, in the second interview athletes reflected upon the effects of the whole relocation process on their sport, personal, and vocational development. In general, all players believed that migration had strongly improved their sport career opportunities, being a springboard towards handball professionalization. Furthermore, a consensus emerged in relation to the relevant visibility of being national team players in creating migration opportunities and a direct recruitment from a foreign sport club.

*“At the beginning of my career, I decided I would do everything to succeed. I would not return home as a loser. I wanted to be a winner. Not because of my father but because of myself”.*

(SLO player)

*“Wer es nicht probiert, hat schon verloren. [Who hasn’t tried has already lost]”.*

(SUI player)

All participants reported that they considered dual career an essential aspect of their migration choice, being part of their plans from the very beginning of this experience. In particular, all the athletes mentioned that their families demanded them to complete their education while pursuing their international athletic goals. To note, one SLO player declared that her athletic migration experience gave her dual career a special push. Interestingly, the majority of the athletes showed an interest in working in either sports management or some other sport-related business after finishing their handball careers.

*“Switzerland was not “big enough” for my sport career dreams. In pursuing my goal to play my sport professionally, I wanted also to check how the other European countries were organizing sport in combination with academic studies. I decided to study sport in Denmark, the “heart of women’s handball”. I was ambitious in both sport and in my other career”.*

(SUI player)

*“My family was repeating me that ‘It is very nice that you are enjoying handball, but school comes first!’”*

(SUI player)

*“When I was at home, I expected my mother to remind me about school. Now I know that I somehow considered my school as my mother’s business, and not mine. When I moved abroad, I finally realized that everything in my life is my responsibility, including my education”.*

(SLO player)

All the participants believed that transnational mobility contributed to their personal growth, offering them the possibility to develop life competencies and skills (i.e., decision-making; time management; goal orientation; teamwork; communication; self-organization; and responsibility). Furthermore, the participants mentioned also the benefits deriving from athletic migration for acquiring vocational skills, learning foreign languages, expanding their social networks, and ameliorating their general lifestyle through different sociocultural perspectives.

*“The biggest lesson was to have the self-confidence to arrive as a Swiss player into a very big handball nation and show them that we are able to play handball as well. A positive mind-set and psychology, combined with self-confidence, were very important in the first three years. In the last two years, I got used to it and I played better and better because I felt secure and confident. I also learnt that without self-confidence I would never have been a good player. I believed in my strength and made my own way. This was a lesson for life”.*

(SUI player)

*“Germans are different, so structured and disciplined. Conversely, Slovenians are part of the Balkans, more creative and passionate, and looking for better life prospects”.*

(SLO player)

*“I currently work for the Handball Federation. The job is very interesting and also nice for me because I know more or less how everything works from my experience as a player. My time playing in other countries also helped. I speak fluent English and I had chance to gain experience regarding other handball cultures, such as Danish, Slovenian and German. People I work with maybe still know me and accept me as a foreign professional player”.*

(SUI player)

*“French (language) would be an important competence in my post-handball career”.*

(SLO player)

All the athletes admitted having experienced some difficulties during the pre-transition and acute cultural adaptation phases. Indeed, SLO players described their first migration as a big, difficult, and stressful step in their careers, but also as the best decision of their life. After having adapted to the new socio-cultural environment, they perceived they had higher sport results and an overall enriched life experience with respect to other not-migrating athletes. Whilst the SLO players reported that their migration experience was meant to build potential future life and occupational prospects in the relocation countries, the SUI player expressed no doubts about returning to Switzerland in the future.

*“Going abroad was the best decision in my life. I enjoy playing handball, I’ve got a good job and I found love—I got married here”.*

(SLO player)

*“I knew from the beginning of my migration experience that my future would be in Switzerland. After my handball career, I intended to return to Switzerland, and I had a clear future plan to return to Switzerland to work. Occupational and opportunities in life are too excellent in Switzerland to stay elsewhere”.*

(SUI player)

Overall, in considering the conceptual categorization of student-athlete migration the thematic analysis identified four main-level constructs [49]: (1) ambitious, mainly for sport achievements); (2) nomadic cosmopolitan, with SLO players valuing different cultural experience in several EU countries; (3) returnee, with SUI players relocating in their native country at the end of their competitive career abroad; and (4) settler, SLO players considering remaining in a host country beyond their sport career.

## 4. Discussion

Considering that previous scientific studies on dual career migration mainly focused on male athletes with a final destination in several countries worldwide [3], the longitudinal approach of the present study contributes new knowledge to literature on female athletic migration. Therefore, it was deemed appropriate to investigate the mobility of international dual career female handball players, as border-crossing occurred within Europe where dual careers are still at a developmental stage [10,11,45,49]. The main findings of the present work highlighted that female handball players succeeded in combining their academic and sports paths. Despite participants reporting several challenges prior and during their migration, they considered their transnational experiences positive overall.

In examining the push factors of dual career migration of SLO and SUI female players, it is relevant to consider that some European countries attract athletes wishing to experience higher sports achievements in countries having a high international role in women’s handball ranking [62]. In the last decade, it has become common to acknowledge that achieving a highly successful career in elite female handball is synonymous with playing at the highest international level, putting a sporting migrant in a prominent sporting context. Athletic migration has intensified worldwide, also intensively impacting women’s handball [2,3,4,5,6,7,28,39]. The present research focused on the migration of elite national handball players from small European countries with a low handball tradition (i.e., SLO and SUI), and on the identification of specific stressors and challenges for migrant athletes, such as dual career challenges, and cultural, linguistic, and structural barriers underpinning athletic relocation [34]. According to the sport globalization phenomenon, the participants to this study mainly migrated to Denmark, France, Germany, Hungary, Montenegro, Norway, Romania, Spain, Macedonia, Poland, and Croatia, in which handball is highly developed at club level through extended networks on an international scale, providing also financial support to female players [4,28,47,52]. In particular, SUI players tended to migrate to German language countries, whereas SLO players resulted more dynamic in their migration presenting a high number between countries and clubs transfers. In particular, for SUI athletes, the development of their handball skills and opportunity to gain new life experiences were considered the most crucial determinants of their athletic migration. Conversely, SLO players considered the advancement in their sports careers together with the development in their personal sphere (such as cultural enrichment and gaining independence from their families) as key priorities for athletic migration. These results are confirmed by previous research on European migrant athletes [5,26,33], which revealed that athletes’ career trajectories, life experiences, and psychosocial functioning were closely linked to career discourse practices, professional opportunities, and social migration policies in their native country.

Differently from the literature, no mercenary profile emerged in this study, probably because the financial revenues provided to the female handball players were not sufficient to capitalize for future years [3,5,50]. Despite appreciating the financial benefits deriving from their migration, the interviewed players stressed the expectation of athletic and personal growth as the main reason for relocating abroad. According to the literature [5,9,30,31], athletes consider the high-level club environment, the professional coaching, the interaction with expert teammates, and the competition in prestigious handball national championships to be important factors in their sport development. In this framework, sport migration is crucial for their success in their handball career and personal growth [7]. In fact, the participants of this study declared that their migrating experience increased their sport-related skills, training and competition conditions, professional status, and international visibility, thus representing the ambitious category of migrating athletes [50].

The ambitious category could not be considered a separate category when overlapping with other push factors of the athlete’s personal life [5,41]. In appreciating their unique sport and cultural experience abroad as valuable for post-career transition to the labor market, the players could be also included in the nomadic–cosmopolitan category [50]. In this respect, the players highlighted the relevant support they received from their parents to migrate. According to the literature [5,17,63,64,65,66], parental beliefs, expectations, behaviors, and social support play a relevant emotional, motivational, instrumental, material, and social role in the development of dual career athletes, including the student athletes’ decision to select a specific academic institution and/or to look abroad to pursue their academic and sport careers. Despite the availability of educational opportunities abroad, considered a push factor, only one SUI player decided to migrate to Denmark for academic purposes, whereas the rest of the participants maintained their academic commitments through distance learning. All participants confirmed essential dual careers opportunities as a migration outcome [2,3,5,21,47,48,49]. Interestingly, one SLO player declared that migration enhanced her motivation to pursue a dual career. In considering that all athletes met goals for their academic degrees in combination with their sports careers abroad, these findings substantiate the crucial roles that e-learning and academic flexibility play in guaranteeing adequate support and care of elite student athletes [44,46].

Despite the fact that players considered migration experience valuable at a personal level, they also reported difficulties in developing a sense of belonging at the beginning of their migration process. In the literature, some athletes complained about the complexity of their cross-cultural experiences, inequality in opportunities, and the difficulty of developing a sense of belonging [5,9,27,34]. In the present study, the players adopted different coping strategies and received the support of other club members, which helped them to adapt to the new environment quickly [34,36,37]. In particular, current and former club and national team teammates provided support in the first phase of the migration process, and in some cases represented key facilitators for enabling and organizing the whole migration process through their networks with coaches or clubs, and the provision of contacts, as well as improving the quality of migrant athletes’ social life outside the sport environment. Finally, participants with nearly a decade of migration experience also claimed that through hard and dedicated work they achieved a good position in the club, felt valued, enriched their social relationships, and enlarged their professional network. In addition to handball-related aspects, in the adaptation to the hosting countries, SUI and SLO migrant players reported learning a new language (i.e., especially SLO players) or adjusting to different cultural norms as additional challenges [4,5,33]. All participants in this study mentioned the benefits of acquiring some specific occupational skills and competences, expanding their social networks, learning some essential life skills together with a new lifestyle and with different sociocultural perspectives.

In general, the players considered their careers successful, being satisfied with their migration decision, especially when comparing their careers with those of non-migrating counterparts. At the end of their handball career, the majority of players tended to return to their native country, substantiating the returnee category of migrating athletes [39,50]. The SLO players tended to continue playing in a foreign country, accumulating 8–12 years of migration experience and a cross-cultural adaptation. They also pointed out the benefits of knowing different cultures, gaining excellent life experience, developing new skills and competences, and reporting the intention to settle abroad for their post-athletic career transition as settlers [50]. Thus, the long-term migration of SLO players represents a new trend of post-handball career retirement of female players [39].

## 5. Conclusions

The present study generated new evidence for more structured and effective career development paths in women’s elite handball. In particular, sport migration from geographically small countries presenting limited sport opportunities could be considered as a key career determinant. In fact, all the participants of this study highlighted the benefits derived from sport relocation for their career success, especially in considering the limited sport opportunities available in their native countries. Furthermore, the development of their life and vocational skills and competencies has been identified as a crucial determinant when considering athletic relocation. In exploring the role of migration in the sport career, the characteristics of the athletic relocation, the athletes’ reasons and/or goals for migration, the dual career strategies, the coping process with career and migration barriers/challenges, and the competence/skill development during the migration process, the present findings could provide some suggestions and recommendations to promote a holistic development for migrant female handball athletes: (1) the possession of excellent handball skills and a clear intention towards building a professional handball career; (2) the actual fulfilment of professional handball career aspirations; (3) the planning of clear dual career goals at the beginning of the migration process; (4) high personal ambition and emotional connection to handball; (5) the implementation of a successful dual career path; (6) a positive migration experience; and (7) feeling supported and valued in the new team and community environments.

Thus, the career trajectory of women elite athletes migrating in order to accomplish higher sport goals should encompass multidimensional developmental factors (i.e., athletic, educational, vocational, and personal) to promote a holistic development of the athlete as an individual and to facilitate career transitions and post-athletic career adjustments. In this framework, dual career opportunities (i.e., at educational and/or vocational level) are a crucial determinant to provide the athletes with additional relocation benefits and to equip them with necessary skills and competencies to prepare for post-sport retirement. This aspect is important for sportspeople at large, independently from the athletic domain. However, in minor sports and for athletes migrating from countries with limited athletic growth possibilities, the enrichment of their relocation experience (i.e., which may last many years) becomes even more important for both their personal and professional growth. Indeed, many former migrant players used their international experience to enroll in the sporting job market, contribute to women’s handball development as managers or team officials within handball national teams/federations, and/or to professionally relocate outside the sport environment, turning into vocational migrants. Therefore, sport federations and elite sport clubs recruiting migrating athletes should be sensitized towards successfully addressing these issues to facilitate the transnational relocation of elite female athletes. In particular, providing athletes with necessary support and opportunities may contribute to both enrich their migrating experience, to increase their sport performances, and to ameliorate their socio-cultural integration. Although some factors might be generalized, it is important to emphasize that each migration story is unique.

The limited number of athletes accomplishing the whole duration of the research and the involvement of athletes from two countries could be considered limitations of this study. To further explore career trajectories and choices of migrating female athletes, future studies should envision the involvement of a higher number of participants from different countries, representing a variety of sport disciplines. This approach would help to identify significant and specific socio-cultural and personal characteristics/determinants/predictors to facilitate successful transnational migration and dual career development of elite athletes. Despite the high reputation of the research team members on dual career of sportspersons and international handball, the lack of an external piloting phase including external experts could represent a further limitation of the study.

## Figures and Tables

**Table 1:** Thematic analysis of career/life development and athletic migration.

Thematic Domains	Subthemes		
	Before Migration	During Migration	After Migration
**Dual career** **(Sport and** **Academic** **Development)**	Decision to pursue a professional sport career	Organisation of the transnational elite sport career	Evaluation of the sport career success
	Elite athlete status in the native country	Sport achievements in relocation countries	Assessment of educational performance after migration
	Experiences in domestic handball clubs	Coping with transnational elite sport career transitions	The role of migration in dual career development
	The role in the national team	Relationships with the new club, coaches and other players	
	Evaluation of the sport career in the native country	Organisation of dual career	
	Evaluation of the educational status in the native country	Coping with dual career challenges	
	Evaluation of the dual career in the native country	Educational achievements during migration	
**Personal/** **Life Sphere**	Personal development	Influence and support of family/peers and sports agents	Role of migration in personal development
	Expectations from cross-national mobility	Life skills development during migration	Role of migration in creating vocational opportunities
	Family/peers role in the decision to migrate		Future career plans
	Professional motivations to migrate		
**Migration**	Reasons to migrate	Coping	Evaluation of the whole migration process
	Organisation of transnational migration	Barriers	
	Expectations from cross-national mobility	Adaptation strategies	
		Changes in the social environment	

**Table 2:** Participants’ information regarding their handball career development before 2012.

Descriptor	SUI (*n* = 6)	SLO (*n* = 8)
Age at start playing handball	8 years old	6 years old
Facilitators to start playing handball	Parents/Friends	Elementary school teacher, Coaches, Parents/Friends
Handball status in the native country	Amateur	Semi-professional
Number of domestic relocations prior to transnational migration	33% players; 1 relocation	100% players; range: 1–5 relocations
Number of years playing at the senior level in the native country	Average: 4.7 yr; range: 2–7 yr	Average: 5.4 yr; range: 2–8 yr
Age in 2012 (“Interview 1”)	26.3 ± 3.1 yr; range: 20–27 yr	24.7 ± 2.6 yr; range: 21–26 yr
Years spent abroad prior to 2012	2.1 ± 0.5 yr; range: 2–4 yr	3.9 ± 0.2 yr; range: 2–8 yr

**Table 3:** Participants’ athletic and migration features during the study course (2012–2019).

Nationality	Years Competing (*n*)	Migration Years (*n*)	Migration Countries (*n*)	Still Active in 2019	Still Migrant in 2019
SUI	4 out of 7	1 out of 4	(1) Germany	No	No
SUI	5 out of 7	2 out of 5	(2) Germany, Slovenia	No	No
SUI	6 out of 7	5 out of 6	(1) Germany	No	No
SUI	7 out of 7	7 out of 7	(2) Germany, Denmark	Yes	Yes
SUI	7 out of 7	6 out of 7	(2) Germany, Norway	Yes	No
SUI	7 out of 7	2 out of 7	(1) France	Yes	No
SLO	2 out of 7	2 out of 2	(2) Spain, Turkey	No	No
SLO	7 out of 7	7 out of 7	(3) Germany, Hungary, France	Yes	Yes
SLO	7 out of 7	2 out of 7	(1) Spain	Yes	No
SLO	7 out of 7	7 out of 7	(3) Montenegro, Macedonia, Romania	Yes	Yes
SLO	7 out of 7	6 out of 7	(3) Germany, Spain, Poland	Yes	Yes
SLO	7 out of 7	4 out of 7	(1) France	Yes	No
SLO	7 out of 7	7 out of 7	(3) Montenegro, Croatia, Switzerland	Yes	Yes
SLO	7 out of 7	7 out of 7	(2) Germany, Spain	Yes	Yes

## Data Availability

Data supporting the present study are anonymously archived in the University of Ljubljana.
